# The role of EphA2 in ADAM17- and ionizing radiation-enhanced lung cancer cell migration

**DOI:** 10.3389/fonc.2023.1117326

**Published:** 2023-03-14

**Authors:** Verena Waller, Fabienne Tschanz, Rona Winkler, Martin Pruschy

**Affiliations:** Laboratory for Applied Radiobiology, Department of Radiation Oncology, University Hospital Zurich, University of Zurich, Zurich, Switzerland

**Keywords:** ionizing radiation, EphA2, ADAM17, Ephrin-A1, cancer cell migration

## Abstract

**Purpose:**

Ionizing radiation (IR) enhances the migratory capacity of cancer cells. Here we investigate in non-small-cell-lung-cancer (NSCLC) cells a novel link between IR-enhanced ADAM17 activity and the non-canonical pathway of EphA2 in the cellular stress response to irradiation.

**Methods:**

Cancer cell migration in dependence of IR, EphA2, and paracrine signaling mediated by ADAM17 was determined using transwell migration assays. Changes of EphA2 pS897 and mRNA expression levels upon different ADAM17-directed treatment strategies, including the small molecular inhibitor TMI-005, the monoclonal antibody MEDI3622, and shRNAs, were mechanistically investigated. ADAM17-mediated release and cleavage of the EphA2 ligand ephrin-A1 was measured using ELISA and an acellular cleavage assay.

**Results:**

Irradiation with 5 Gy enhanced tumor cell migration of NSCLC NCI-H358 cells in dependence of EphA2. At the same time, IR increased growth factor-induced EphA2 S897 phosphorylation *via* auto- and paracrine signaling. Genetic and pharmaceutical downregulation of ADAM17 activity abrogated growth factor (e.g. amphiregulin) release, which reduced MAPK pathway-mediated EphA2 S897 phosphorylation in an auto- and paracrine way (non-canonical EphA2-pathway) in NCI-H358 and A549 cells. These signaling processes were associated with reduced cell migration towards conditioned media derived from ADAM17-deficient cells. Interestingly, ADAM17 inhibition with the small molecular inhibitor TMI-005 led to the internalization and proteasomal degradation of EphA2, which was rescued by amphiregulin or MG-132 treatment. In addition, ADAM17 inhibition also abrogated ephrin-A1 cleavage and thereby interfered with the canonical EphA2-pathway.

**Conclusion:**

We identified ADAM17 and the receptor tyrosine kinase EphA2 as two important drivers for (IR-) induced NSCLC cell migration and described a unique interrelation between ADAM17 and EphA2. We demonstrated that ADAM17 influences both, EphA2 (pS897) and its GPI-anchored ligand ephrin-A1. Using different cellular and molecular readouts, we generated a comprehensive picture of how ADAM17 and IR influence the EphA2 canonical and non-canonical pathway in NSCLC cells.

## Introduction

Lung cancer is the second most frequent type of cancer in men and women and has with 18%, the highest cancer-related mortality worldwide (1). Current first line treatment strategies comprise either radiotherapy (RT) alone or in combination with surgery, chemotherapy and/or immunotherapy (2). On the molecular level, ionization radiation (IR) causes DNA double-strand breaks that induce genomic instability and eventually drive the cells into mitotic catastrophe, apoptosis, or senescence (3). In addition to DNA damage, IR also affects intra- and intercellular signaling processes that trigger a multilayered stress response and can modulate the architecture of the tumor microenvironment (TME) (4). These processes can lead to enhanced radioresistance, immunomodulation, and even tumor cell dissemination and thereby influence clinical outcome (4).

The reprogramming of the epithelial-mesenchymal transition (EMT) by IR has been described as one of the hallmarks determining treatment success (5–7). Therefore, it is important to understand the processes that contribute to EMT to identify treatment strategies that can overcome radioresistance. The Eph (erythropoietin-producing hepatocellular) receptor tyrosine kinase family, which is divided into nine EphA and five EphB receptors, and their respective ligands (ephrin-A and ephrin-B) have been implicated as important drivers for tumor promotion and cell migration by their direct and indirect crosstalks with different EMT markers, such as E-Cadherin, N-Cadherin and Snail (8). One of its best-described members, EphA2 has gained increasing attention due to its ligand-dependent (canonical) and ligand-independent (non-canonical) signaling that can either display a tumor suppressive or oncogenic behavior, respectively. The GPI-anchored ligand ephrin-A1 activates EphA2 through autophosphorylation on its tyrosine residues, which attenuates oncogenic Ras/MAPK signaling (9). In the absence of direct ligand interaction, EphA2 is phosphorylated on serine residue 897 by other growth factor-stimulated receptor tyrosine kinases (RTKs) and their respective downstream signaling. This activation step eventually leads to a migratory and invasive phenotype of the cell (10). In comparison to its ligand, EphA2 is often overexpressed in various cancer types, in particular non-small cell lung cancer (NSCLC), where it is associated with poor prognosis (11–15). Taking its role in tumorigenesis, tumor cell adhesion, migration, metastatic spread, and angiogenesis into consideration, (non-) canonical EphA2 receptor-mediated signaling represents an important but so far much-neglected cascade to better understand the stress response to irradiation. Interestingly, only few studies investigated EphA2 in the context of ionizing radiation with Graves et al., being the first to link between EphA2 S897 phosphorylation and IR (16–18).

We here describe a unique interrelation between EphA2 and the disintegrin and metalloproteinase ADAM17 alone and in response to irradiation. ADAM17 is involved in the processing of the extracellular matrix and the release of various growth factors and cytokines that contribute to the remodeling of the TME and facilitate tumor cell dissemination (19). We recently have shown that IR enhances ADAM17 activity in NSCLC cell lines which contributes not only to the radioprotection of the tumor itself, but also its vasculature (20, 21). Here, we investigate IR-induced tumor cell motility and link it mechanistically to an IR-enhanced ADAM17-EphA2 signaling network acting on an auto- and paracrine level.

## Materials and methods

### Cell lines

The human lung adenocarcinoma cell lines H358 and A549 (ATCC, Manassas, VA, USA) and the human prostatic adenocarcinoma cell line PC-3 (ATCC, Manassas, VA, USA) were newly purchased and cultured in RPMI1640 media (Gibco; 22409-015), supplemented with 10% (v/v) fetal bovine serum (Gibco; 10270-106), 1% (v/v) penicillin-streptomycin (Gibco; 15140122), and 1% (v/v) L-glutamine (Gibco; 35050-38). The cells were cultured as monolayers in culture plates in a 37°C humidified atmosphere containing 5% CO_2_.

### Stable cell lines

Custom lentiviral shRNA expression vectors were obtained from Cellecta. The expression vector used for shRNA directed against ADAM17 (A17) and control (NT) was pRSITPRP-U6Tet-sh-PGK-TetRep-2A-TagRFP-2A-Puro. Insert sequence for shA17 was ACC GGG ATC ATC GCT TCT ATA GAT ACG TTA ATA TTC ATA GCG TAT CTG TAG AAG CGA TGA TCT TTT and for the control cell line shNT was ACC GGC AAC AAG ATG GAG AGC ACT AAG TTA ATA TTC ATA GCT TGG TGC TCT TCA TCT TGT TGT TTT. The expression vector used for shEphA2 and shScr was pLKO-Tet-On. Insert sequence for shEphA2 was 5’-CCG GCG GAC AGA CAT ATA GGA TAT TCT CGA GAA TAT CCT ATA TGT CTG TCC GTT TTT-3’, and for the control cell line shScr was 5’-CCG GGA CAC GTC AAC TCG AAT AAC TCT CGA GAG TTA TTC GAG TTG ACG TGT CTT TTT-3’. The pLVX lentiviruses encoding FLAG-tagged EphA2 wt, and FLAG-tagged EphA2 S897A were kindly provided by E. Pasquale (Sanford Burnham, Prebys Medical Discovery Institute, La Jolla, CA) (22).

The lentiviral constructs were packaged into pseudotyped viral particles in HEK293AD cells, using the psPAX2/pMD2.G packaging plasmid mix (Cellecta, #CPCP-K2A) according to the manufacturer’s protocol. Target cells were transduced with the lentiviral supernatants in presence of 5 µg/ml polybrene. Cells were selected in presence of 1 µg/mL puromycin. The induction of the directed shRNA construct always began 72h prior to experiment start with 1 µg/ml Doxycycline (Sigma; D9891).

### Cell treatment and irradiation

MEDI3622 and IgG1 were directly obtained from AstraZeneca. Cells were pretreated with MEDI3622 or IgG1 24 hours prior to sham treatment or irradiation (5 Gy) with a RS-2000 225kV irradiator at 4.2 Gy/min (Rad Source).

The small molecular inhibitors TMI-005, SCH772984, and MG-132 were obtained from Axon Medchem (Axon 1507), Biovision (B1687) and Sigma (M7449), respectively. The human recombinant ligands rhEGF and rhAmphiregulin were obtained from Sigma Aldrich (E9644) and Genescript; (Z03103-50). rhEphrin-A1 Fc was obtained from R&D Systems (#6417-A1-050). Cells were treated as described in the results section. For sham treatment PBS or DMSO were used.

### Transwell migration assay

For the transwell migration assay, 24-well transwell units (6.5mm diameter) with 8μm pore size PET membranes (Greiner Bio-One; 662610 and 662638) were used according to the manufacturer’s instructions. Briefly, for the generation of the conditioned media (CM) 4.0 x 10^5^ cells (H358 NT, H358 A17) were plated into a 6-well plate in 2ml full RPMI. Simultaneously, 3 x 10^4^ migrating cells (H358 shScr, H358 shEphA2) were seeded into transwell inserts (“upper chamber”). On the next day, cells were starved in either RPMI supplemented with 1% FBS (6-well plates) or 0.5% FBS (cells in the inserts). Six hours later the inserts and plates were sham-irradiated or irradiated with 5Gy. 24h post-irradiation, the CM from the 6-well plates containing sham-irradiated or irradiated H358 NT, or H358 A17 was transferred into 24-well plates (“lower chamber) and the inserts were immediately placed on the wells harboring the attracting media. The co-culture was maintained at 37°C in 5% CO_2_ for 24hours. For quantification, cells from the upper side of the insert were scraped away with a cotton swab and inserts were then fixed in Methanol/Acetic Acid (75%/25%, v/v), dried and stained with DAPI (Invitrogen; D1306, 1:25’000) in 99% MeOH. Fluorescent microscopy pictures were taken (Leica 7000DT) and the migrated cells were counted manually in at least 3 images at 10x magnification per insert.

### Immunoblotting and antibodies

For the detection of proteins in cell lysates, cells were lysed with M-PER™ Mammalian Protein Extraction Reagent (Thermo Fisher; 78501) supplemented with 1x Halt™ Protease and Phosphatase Inhibitor Cocktail (Thermo Fisher; 78440). 30µg of protein lysate was used per lane in the SDS-PAGE and after western blotting the membrane was blocked for 20’ in EveryBlot Blocking Buffer (Bio-Rad, 12010020). For the assessment of protein expression, the following primary monoclonal antibodies (1:1000 dilution) were purchased from Cell Signaling Technology: rabbit anti-EphA2 (#6997), rabbit anti-phospho-EphA2 (Ser897) (#6347), rabbit anti-phospho-EphA2 (Tyr772) (#8244), rabbit anti-ERK1/2 (#4695S), rabbit anti-phospho-ERK1/2 (Tyr202/Tyr204) (#4370S), rabbit anti-AKT (#9272), rabbit anti-phospho-AKT (Ser473) (#9271S). Protein bands were visualized using an anti-rabbit horseradish peroxidase (HRP)-coupled secondary antibody (*Santa Cruz* Biotechnology; sc-2357) (1:5000 dilution) and enhanced chemiluminescence (Amersham; RPN2232). For the loading control mouse anti-alpha Tubulin HRP (Genetex; GTX628802-01) (1:5000 dilution) was used.

### qRT-PCR

Sample mRNA expression was determined 24 hours after treatment, if not specified otherwise. Cell lysate collection and RNA isolation were performed using a RNeasy Mini Kit (Qiagen; 74004) according to the manufacturer’s instructions. RNA was reverse transcribed using High-Capacity cDNA Reverse Transcription Kit (Applied Biosciences; 4368814) and cDNA was amplified using SYBR Green Master Mix (Sigma; S9430) with the following primers from Microsynth: (5′-3′): GAPDH forward: AACGGATTTGGTCGTATTGGGC; GAPDH reverse: TTGATTTTGGAGGGATCTCG; EphA2 forward: GGGACCTGATGCAGAACATC; EphA2 reverse: AGTTGGTGCGGAGCCAGT. qRT-PCR was performed on LightCycler 480 (Roche).

### Immunofluorescence and antibodies

NCI-H358 cells were seeded 24h prior the experiment onto a 6-well plate with cover glasses. The cells were treated with 25µM TMI-005 or DMSO for 8h, 16h and 24 or with rhEphrin-A1 Fc (1µg/ml) for 20min. After the respective incubation times, the cells were washed with ice-cold PBS and fixed in 2% formaldehyde/PBS for 15min at RT. The cells were washed three times with PBS-T (0.02% Tween-20 in PBS) and permeabilized with 0.5% Triton X-100/PBS for 10 min. After washing, the cells were blocked 2% BSA/PBS-T for 1 h. Then cells were incubated with the primary antibody rabbit anti-EphA2 (1:200 in 2% BSA/PBS-T, Cell Signaling Technology; #6997) or an IgG1 control (AstraZeneca) at RT for 1h in a humidified chamber. After six times washing with PBS-T, the cells were incubated with secondary antibody goat anti-rabbit Alexa Fluor488 (1:200, Invitrogen; A11070) and DAPI (1:25000, Invitrogen; D1306) for 1 h at room temperature, in a dark, humidified chamber. After six times washing with PBS-T and two washings with PBS, the cover glasses were mounted on microscope glass slides with Dako fluorescence mounting medium (Agilent; #S3023). The microscope images were acquired on Leica DMi8 microscope using a HC PL APO CS2 63x objective, a Leica digital color camera DMC6200 and Leica monochrome fluorescence DFC9000 GTC using 390 and 475 nm laser lines. Refractive index-matched immersion oil (Nikon Instruments) was used for all experiments. Images were reconstructed and analyzed with FlowJo software.

### ELISA

200’000 NCI-H358 cells were seeded 24h prior the experiment onto a 6-well plate. Soluble human Ephrin A1 was collected 24 hours and 48 hours after treatment and detected in 200µl filtered conditioned media using a human Ephrin-A1 ELISA kit and a human Amphiregulin DuoSet ELISA kit according to the manufacturer’s guidelines (Chemie Brunschwig AG; HUEB1504 and R&D Systems; DY262 respectively). The absorbance was determined with a plate reader TECAN Infinite 200 PRO at 450 nm excitation and 540 nm emission and normalized to the total protein amount of the cell lysate.

### Acellular cleavage assay

A total of 100 ng human recombinant EphrinA1-Fc was incubated with 100 ng human recombinant ADAM17 (Sigma-Aldrich; TACE Activity Kit CBA042, kit component no. JA9119) in the TACE Activity Kit Assay buffer (Sigma-Aldrich; TACE Activity Kit CBA042, kit component no. JA9121) in a total volume of 25μl per reaction. The samples were incubated +/- TMI-005 for 5h at 37°C. For Western Blot analysis, the samples were boiled at 95°C in 6x Laemmli Buffer.

### Statistical analysis

The data were analyzed with GraphPad Prism v9 and tested for normal distribution using the Shapiro-Wilk normality test. For the direct comparison of two treatment groups, the two-tailed unpaired t-test or Mann-Whitney test was applied. For the comparison of more than two treatment groups one-way ANOVA test with Tukey posttest or Dunn’s test was performed. Error bars indicate SEM. A p-value ≤ 0.05 was considered significant. *If not indicated otherwise*, data were collated from at least three biological replicates obtained from at least three independent experiments. For all experiments applies; p<0.05: *, p<0.01: **, p<0.001: ***.

## Results

### IR-Induced cancer cell migration is dependent on EphA2

**Figure 1:**
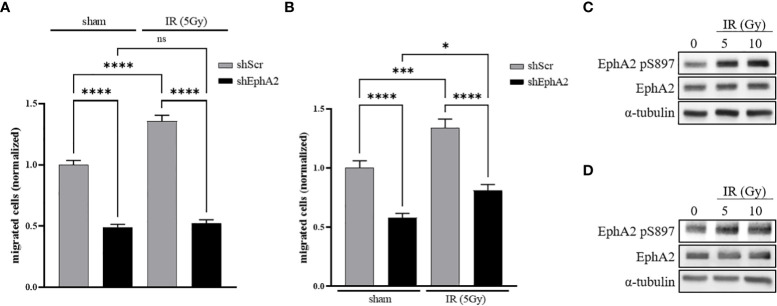
IR-induced cancer cell migration is dependent on EphA2. **(A)** A doxycycline-inducible shRNA system directed against EphA2 (shEphA2; or scrambled control, shScr) was used to assess the effect of IR and EphA2 downregulation on the migratory capacities of NCI-H358 tumor cells. **(B)** CM derived from irradiated and sham-irradiated control cells were used to attract NCI-H358 cells in dependence of their EphA2 status (shScr and shEphA2, respectively). **(C)** Increase in EphA2 pS897 in NCI-H358 cells 48h after irradiation with 5 and 10 Gy. **(D)** Increase in EphA2 pS897 in naïve NCI-H358 after incubation with CM derived from sham-irradiated and irradiated NCI-H358 cells. Bar graphs represent the average number of migrated cells counted in three randomly chosen fields/transwell of three independent experiments. *, *P* < 0.05; ***, *P* < 0.001, ****, *P* < 0.0001. ns, not significant.

In order to investigate the relevance of EphA2 for basal and IR-induced lung tumor cell migration, a Boyden-chamber transwell migration assay was developed with NCI-H358 lung adenocarcinoma cells, stably transduced with a doxycycline-inducible EphA2-directed shRNA (shEphA2) vector or an inducible scramble shRNA (shScr) expression vector (**Figure S1A**). Doxycycline-treated cells were seeded into the inserts and starved in 0.5% FBS containing cell growth media. Cell migration towards growth media supplemented with 1.0% FBS was quantified 48 hours following sham-irradiation or irradiation of the inserts with the sublethal dose of 5 Gy (21). Basal cell migration of NCI-H358 control cells was enhanced in response to irradiation, while basal and IR-enhanced migration was strongly abrogated in EphA2 knockdown cells (**Figure 1A**).

To differentiate between an auto- and paracrine effect of IR-enhanced migration, non-irradiated NCI-H358 cells were seeded into inserts and attracted by conditioned media (CM) derived from NCI-H358 cells sham-irradiated or irradiated 24 hours prior to CM transfer. A significantly enhanced increase of cell migration could be determined towards the CM derived from irradiated cells. Migration was again strongly abrogated upon downregulation of EphA2 in the migrating cell population (**Figure 1B**).

Growth factor-induced EphA2 S897 phosphorylation was previously identified as a key driver for cell migration and invasion (10). We therefore investigated the phosphorylation status of EphA2 S897 in response to IR and determined increased EphA2 pS897 phosphorylation 48h following irradiation of NCI-H358 wt cells by 129% (5 Gy) and 150% (10 Gy) (**Figure 1C**). We could also identify a paracrine effect on EphA2 S897 phosphorylation when incubating naïve cells with CM derived from irradiated cells by 168% (5 Gy) and 123% (10 Gy) (**Figure 1D**). These results suggest that irradiation induces the release of soluble and pro-migratory factors that increase EphA2 pS897-mediated cancer cell migration.

### ADAM17 activity regulates EphA2-mediated cell migration through auto- and paracrine signaling

ADAM17 is a major sheddase of multiple growth factors, which may induce RTK-mediated signaling towards EphA2 S897 phosphorylation. To investigate a putative signaling link between EphA2 and ADAM17-mediated tumor cell migration, we first determined the phosphorylation status of EphA2 S897 after pharmaceutical inhibition of ADAM17 activity.

**Figure 2:**
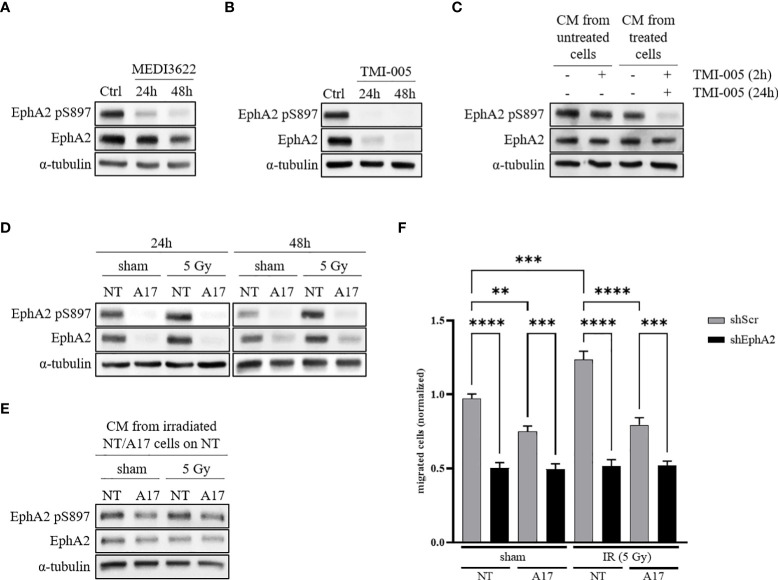
ADAM17 activity regulates EphA2 pS897 and total EphA2 levels through auto- and paracrine signaling. **(A)** ADAM17 inhibition with the mAb MEDI3622 (200nM) abrogates EphA2 pS897 while **(B)** TMI-005 (25µM) additionally reduces total EphA2 levels. **(C)** CM derived from untreated and TMI-005 (24h) treated NCI-H358 cells was transferred on naïve NCI-H358 cells to assess the paracrine effect on EphA2 S897 phosphorylation. To exclude the effect of remaining TMI-005 in the CM, cells were additionally pre-treated for 2h with TMI-005 as indicated in the upper treatment row. **(D)** Protein lysates of NCI-H358 tumor cells transduced with a doxycycline-inducible shRNA system directed against ADAM17 (A17) or control (NT) was used to assess the effect of ADAM17 and IR (24h and 48h post-IR) on EphA2 S897 phosphorylation and total EphA2 levels. **(E)** CM derived from irradiated and sham-irradiated NT/A17 cells was transferred on naïve cells for 2h to elucidate the paracrine effect of ADAM17 knockdown on EphA2 S897 phosphorylation. For all experiments, α-tubulin was used as the housekeeping control. These figures are representative of several independent experiments. **(F)** CM derived from NT/A17 NCI-H358 cells was used to assess the effect of ADAM17 for attracting migrating tumor cells. Bar graphs represent the average number of migrated cells counted in three randomly chosen fields/transwell of three independent biological replicates. **, *P* < 0.01; ***, *P* < 0.001, ****, *P* < 0.0001. For all immunoblotting experiments, α-tubulin was used as the housekeeping control. These figures are representative of several independent experiments.

NCI-H358 wt cells were treated for 24 and 48 hours with the ADAM17-directed inhibitory therapeutic monoclonal antibody MEDI3622 (200nM) or the small molecular ADAM17 inhibitor TMI-005 (25µM). EphA2 pS897 and total EphA2 protein levels were downregulated in response to cellular incubation with both types of ADAM17 inhibitors (**Figures 2A, B**); with a stronger reduction of EphA2 total protein levels in the TMI-005 treated cells compared to cells treated with MEDI3622. This differential potency also indicates that the detected shift of EphA2 S897 phosphorylation is not only due to reduced EphA2 protein levels. Similar results were obtained in the lung adenocarcinoma cell line A549 (**Figure S2A**) as well as in the prostatic adenocarcinoma cell line PC-3 (**Figures S2D**, **S2E**). To elucidate the paracrine effect of TMI-005 treatment, we pre-treated cells for 24h with TMI-005 and transferred the CM on naïve cells. After a 2h incubation, we lysed the cells for western blot analysis. The CM derived from ADAM17-inhibited cells was depleted of factors that could induce EphA2 S897 phosphorylation (**Figure 2C**). We could exclude a putative effect of remaining TMI-005 (from the initial incubation period to gain CM) on the naïve cells themselves, when we supplementing CM derived from untreated cells with TMI-005 2h prior to the incubation with the target cells.

To support the critical role of ADAM17 for growth factor-induced EphA2 S897 phosphorylation we investigated EphA2 and EphA2 pS897 in sham-/irradiated ADAM17 (NT) wildtype and ADAM17 (A17) knockdown cells. EphA2 pS897 was increased in irradiated NCI-H358 control cells 24 and 48 hours after irradiation, but strongly downregulated in sham-irradiated as well as irradiated ADAM17 knockdown cells (**Figure 2D**). A similar response pattern could be observed in the lung adenocarcinoma cell line A549 (**Figure S2B**). Of note, in both cell lines reduced EphA2 pS897 correlated with reduced total expression levels of EphA2 in the ADAM17 knockdown cells. A direct or putative off-target effect of ADAM17-directed shRNA on EphA2 could be excluded determining EphA2 expression over time in ADAM17 knockdown cells (**Figure S1B**). To determine IR- and ADAM17-mediated EphA2 S897 phosphorylation on the paracrine signaling level, naïve NCI-H358 were incubated with CM derived from sham- and irradiated ADAM17 wt and ADAM17 knockdown cells. EphA2 pS897 was strongly upregulated in these cells upon incubation with CM derived from sham- and irradiated ADAM17-proficient cells in comparison to cells incubated with CM derived from ADAM17 knockdown cells. These results strongly support an ADAM17-mediated release of factors regulating phosphorylation of EphA2 S897 in a paracrine manner (**Figure 2E**).

Recent evidence points towards a critical role of EphA2 pS897 for ligand-independent, non-canonical signaling and cell migration (10, 22, 23). To determine a putative ADAM17-dependent and IR-enhanced effect also on tumor cell migration, the transwell migration assay was performed with CM derived from sham-irradiated and irradiated ADAM17 proficient (NT) tumor cells and ADAM17 knockdown (A17) cell, respectively. Cell migration of NCI-H358 cells was reduced towards CM derived from sham- and irradiated ADAM17 knockdown cells, and remained strongly reduced, at basal level, in all EphA2 knockdown cells, independent of treatment (**Figure 2F**). These results point towards ADAM17-mediated signaling and ADAM17/EphA2-dependence for IR-enhanced cancer cell migration.

### ADAM17 and IR regulate EphA2 S897 phosphorylation *via* the MAPK pathway

**Figure 3:**
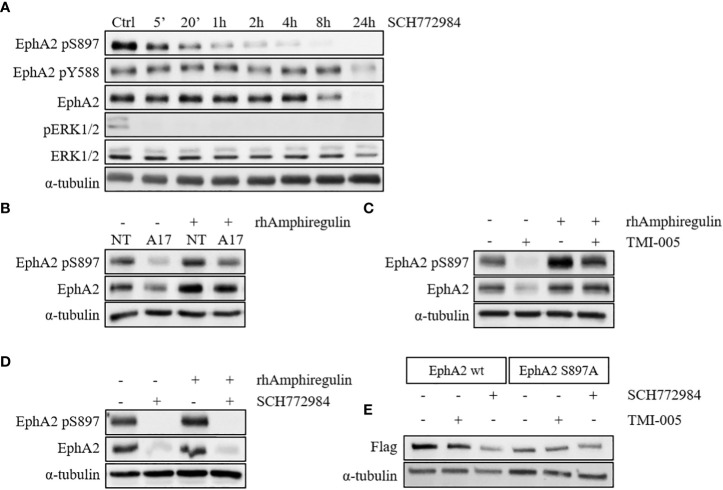
ADAM17 and IR regulate EphA2 S897 phosphorylation *via* the MAPK pathway. **(A)** ERK1/2 inhibition with SCH772984 (500nM) abrogates EphA2 S897 phosphorylation followed by a reduction of the total EphA2 levels. Concurrent rhAmphiregulin (50ng/ml) treatment in **(B)** shADAM17 and **(C)** TMI-005-treated cells rescues EphA2 protein levels, while EphA2 in **(D)** SCH772984-treated cells was not rescued. In all experiments, cells were treated with rhAmphiregulin/TMI-005/SCH772984 for 24h. **(E)** ADAM17 inhibition with TMI-005 reduced flag-tagged EphA2 wt but not the mutated form (S897A). For all immunoblotting experiments, α-tubulin was used as the housekeeping control. The figures are representatives of two independent experiments.

We previously observed a strong IR- and ADAM17-mediated effect on EGFR activation and the downstream MAPK signaling, which could also be corroborated in this study (20). Interestingly prolonged cellular incubation with the small molecular ERK1/2 kinase inhibitor SCH772984 resulted first in rapidly reduced EphA2 pS897, followed by a reduction of the total EphA2 protein levels at later time points (**Figure 3A** and **S2C**). ADAM17 is responsible for shedding the EGFR ligand amphiregulin, thereby activating EGFR downstream signaling (**Figures S3B–F**). Interestingly, ADAM17 inhibition by MEDI3622 only minimally influenced the PKB/AKT pathway (**Figure S3G**). Incubation of either ADAM17 knockdown (**Figure 3B**) or TMI-005 treated NCI-H358 wt cells (**Figure 3C**) with rhAmphiregulin rescued downregulation of EphA2 pS897and the total EphA2 levels. On the other hand, cellular stimulation with rhAmphiregulin did not rescue the inhibitory effect of the ERK1/2 kinase inhibitor SCH772984 (**Figure 3D**). These results suggest that the IR-and ADAM17-regulated secretome activates ERK1/2 kinase upstream of EphA2 pS897 and that the phosphorylation status of EphA2 S897 co-determines the stability of EphA2.

To corroborate the role of ADAM17-mediated EphA2 S897 phosphorylation for the stabilization of the EphA2 protein levels, we generated cell lines with flag-tagged wild-type EphA2 and flag-tagged mutated EphA2 (S897A). Treatment with SCH772984 strongly abrogated total EphA2 levels (anti-flag) in the wt control compared to the S897A mutant (**Figure 3E**). Coherently, TMI-005 treatment only reduced total EphA2 levels in the wt control, while the protein level of mutated EphA2 S897A remained unaffected. These results indicate a critical role for EphA2 pS897 in the ADAM17 (co-) determined stabilization of EphA2. Detailed mechanistic investigations are now required to fully understand the role of this critical S897 residue and the surrounding residues for the stabilization of EphA2.

### ADAM17 activity protects EphA2 from endocytosis and proteasomal degradation

**Figure 4:**
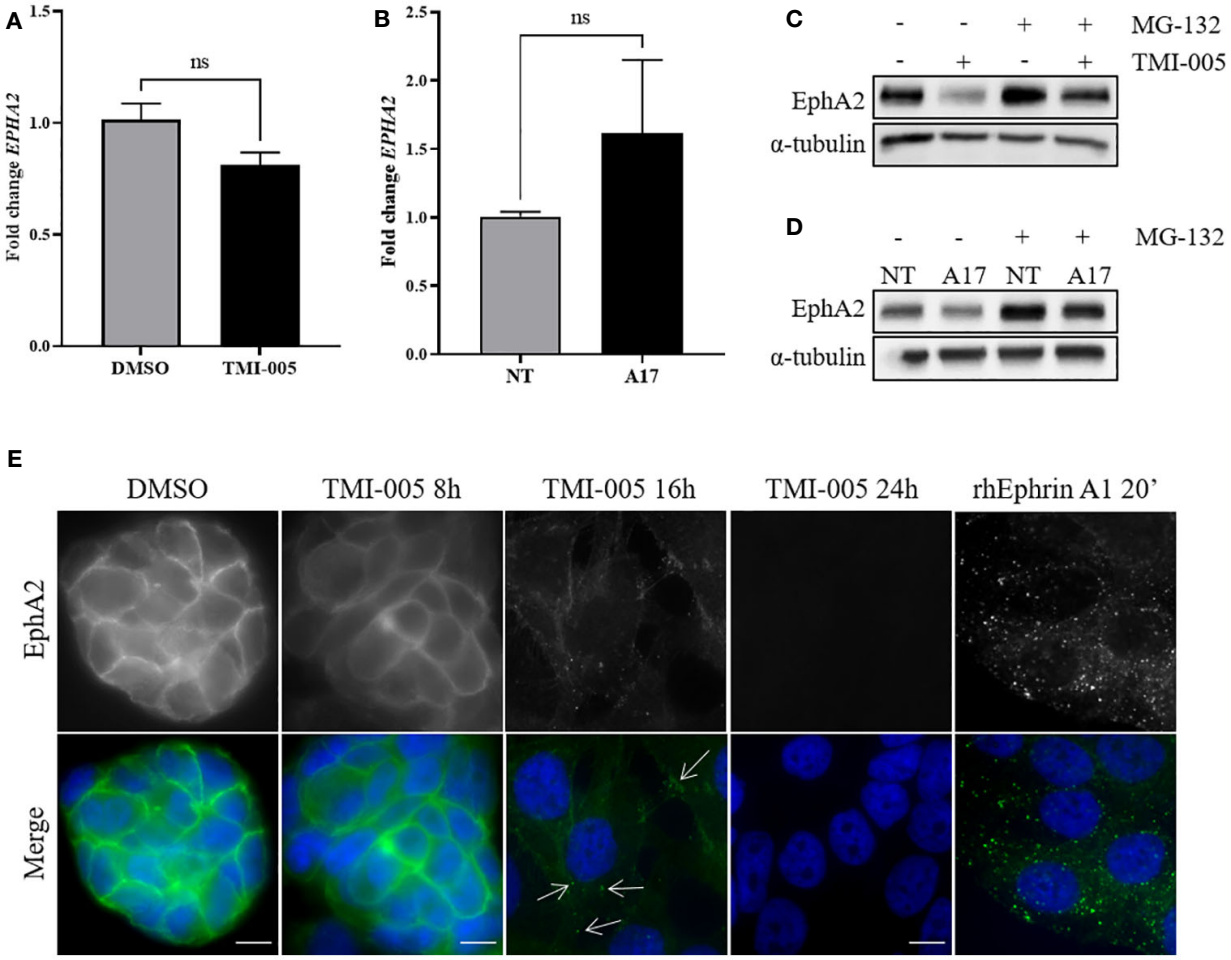
ADAM17 activity protects EphA2 from endocytosis and proteasomal degradation. **(A)** TMI-005 (25µM) or **(B)** ADAM17 downregulation with shRNA directed against ADAM17 (A17) do not significantly alter EphA2 gene expression. Concurrent treatment with MG-132 (1µM) rescues **(C)** TMI-005 and **(D)** A17-induced reduction of total EphA2 protein levels. Cells were treated with MG-132/TMI-005 for 24h. **(A)** and **(B)** Bar graphs represent average EphA2 mRNA expression relative to the housekeeping control GAPDH ± SEM from two independent biological replicates; ns, non-significant. **(E)** The subcellular localization of EphA2 after TMI-005 or upon cellular incubation with rhEphrin-A1 Fc (1µg/ml) was assessed by immunofluorescence microscope. White arrows indicate EphA2 foci. Scale bar = 10µM. These figures are representative of several independent experiments. ns, not significant.

To investigate altered EphA2 protein expression levels in dependence of ADAM17, EphA2 gene expression was determined in naïve NCI-H358 cells treated with TMI-005 and in the ADAM17-directed doxycycline-inducible shRNA knockdown system by qRT-PCR. In contrast to strongly reduced EphA2 protein levels, neither mode of ADAM17 targeting significantly altered EphA2 gene expression (**Figures 4A, B**). On the other hand, cellular pretreatment with the proteasome inhibitor MG-132 restored total EphA2 protein levels in cells treated with TMI-005 or ADAM17 knockdown cells (**Figures 4C, D**).

Membrane-bound EphA2 is tightly regulated through endocytosis. To visualize this process in dependence of ADAM17 activity, EphA2 was fluorescently stained in NCI-H358 cells treated for 8, 16, and 24 hours with TMI-005 (25µM). As control for EphA2 degradation, cells were incubated with the recombinant, homodimeric ligand rhEphrin-A1 Fc, which induces a rapid internalization of the EphA2/ephrin-A1 Fc complex followed by proteasomal degradation (**Figure S4**) (24). As depicted in **Figure 4E**) EphA2 remained membrane-bound for 8h following TMI-005 treatment start. However, at 16 hours, the fluorescence signal shifted into the cytoplasm and was enriched in small foci (as indicated by the white arrows), which is comparable to the staining pattern of cells, treated with rhEphrin-A1 Fc for only 20 min. At the 24h time point, EphA2 could not be detected anymore in TMI-005-treated cells, indicating its complete degradation.

### Ephrin-A1 cleavage is mediated by ADAM17

**Figure 5:**
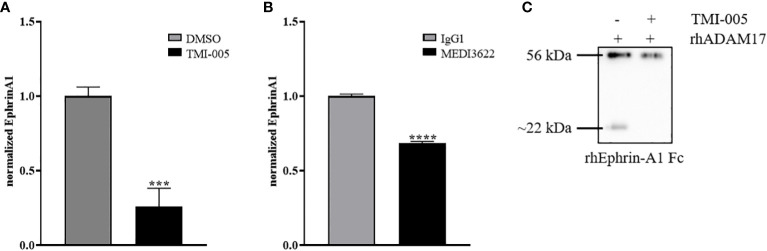
Ephrin-A1 cleavage is mediated by ADAM17. Cellular supernatants derived from NCI-H358 cells were analyzed for cleaved ephrin-A1 24 hours after **(A)** TMI-005 (25µM) or **(B)** MEDI3622 (200nM) treatment by ELISA. **(C)** rhEphrin-A1 was incubated with rhADAM17 for acellular cleavage. TMI-005 inhibited ADAM17-mediated ephrin-A1 cleavage. Bar graphs represent Ephrin-A1 concentration normalized to total cellular protein amount ± SEM from three independent biological replicates. ***, *P* < 0.001; ****, *P* < 0.0001.

To determine the putative contribution of ADAM17 to the release of endogenous ephrin-A1 from the cell surface of tumor cells, we analyzed the supernatant of ADAM17 inhibited cells (TMI-005 or MEDI3622 treated) and compared it to their untreated controls. The level of soluble ephrin-A1 was strongly reduced in NCI-H358 (**Figure 5A**) and PC-3 (**Figure S5**) tumor cells treated with the ADAM17 inhibitor. Likewise, cellular pretreatment of the tumor cells with MEDI3622 abrogated the release of ephrin-A1 into the supernatant (**Figure 5B**). Therefore, we tested whether recombinant human ADAM17 could cleave ephrin-A1 Fc. Indeed, ADAM17 cleaved ephrin-A1 Fc and co-treatment with TMI-005 inhibited ADAM17-mediated cleavage (**Figure 5C**).

## Discussion

We recently reported that IR-induced endothelial cell migration is strongly mediated by tumor cell-located ADAM17 (19, 21). In subcutaneous and orthotopic lung tumor models, ADAM17 inhibition by the novel ADAM17-specific therapeutic antibody MEDI3622 in combination with radiotherapy induced a strong anti-angiogenic effect and tumor shrinkage (21). MEDI3622 was also previously investigated alone and in combination with anti-EGFR–targeting agents in preclinical colorectal and esophageal tumor models demonstrating promising antitumor activity (25, 26). Of interest, MEDI3622 also disrupts ADAM17-mediated immunomodulatory processes (27). Here, we investigated ADAM17 and IR-mediated migration of tumor cells using the lung adenocarcinoma cell line NCI-H358. NCI-H358 cells showed significantly increased migration upon direct irradiation. At the same time, non-irradiated NCI-H358 cells preferably migrated towards conditioned medium, derived from irradiated NCI-H358 cells. This indicates two mechanisms: firstly, that direct irradiation of tumor cells enhances their ability to migrate and secondly, that tumor cell migration is directed, towards an environment enriched with factors released from prior irradiated cells.

ADAM17 was previously identified to play a role in IR-induced cancer cell migration. However, we here demonstrate that ADAM17 also regulates tumor cell migration as part of a paracrine process (28). Our data show, that both, basal and IR-induced cancer cell migration of the lung adenocarcinoma cell line NCI-H358 was reduced towards CM derived from ADAM17 knockdown cells. This indicates that ADAM17 and its shed ligands are driving forces for the directed, IR-induced tumor cell migration.

In addition, we unraveled the unique interrelation between IR, ADAM17, and the essential regulator of cell migration EphA2, whose non-canonical signaling has been described as a key factor for oncogenic cellular behavior and EMT (10, 29). Prior investigations have indicated that EphA2-dependent cell migration is primarily mediated *via* upregulation of typical EMT factors such as N-Cadherin and Snail (29). However, based on more recent evidence we cannot exclude that also amoeboid cancer cell motility is induced (30). ADAM17 and EphA2 have been associated with each other in previous reports, however, a direct regulatory link has not been identified yet (31, 32).

Based on the work of Miao et al. identifying growth factor-induced phosphorylation of EphA2 S897 as a key driver of cell migration and invasion, we here investigated how ADAM17 as a major sheddase of multiple of the aforementioned factors, influences the phosphorylation of EphA2 S897 (10, 26). We demonstrated that ADAM17 regulates (IR-induced) phosphorylation on the relevant serine 897 residue of EphA2 through the MAPK pathway. This is cohesive with a former study by Graves et al., which are one of the very few that have previously investigated the effect of IR on the different EphA2 phosphorylation sites (16). They reported a MEK/ERK/RSK-mediated increase of EphA2 pS897 after irradiation with 2Gy. In addition to this autocrine effect, we could here determine an ADAM17-dependent regulation of EphA2 S897 phosphorylation through paracrine signaling, when incubating naïve cells with conditioned medium, derived from sham- irradiated and irradiated ADAM17 wildtype and ADAM17 knockdown cells. These differential ADAM17-regulated levels of EphA2 pS897 directly correlated with ADAM17-dependent cancer cell migration. Interestingly, when treating NCI-H358 wt cells with the small molecular inhibitor against ERK1/2, SCH772984, we observed a time-dependent decrease in the EphA2 S897 phosphorylation followed by a - delayed - decrease of the total EphA2 protein levels. At the same time, (ADAM17-cleaved) amphiregulin could only rescue reduced levels of EphA2 in response to ADAM17 knockdown or treatment with an ADAM17 inhibitor, but not in cells treated with the ERK inhibitor SCH772984. These results demonstrate that ADAM17 releases factors upstream of RTK-activated signaling to MAPK, which is relevant for EphA2 S897 phosphorylation and EphA2 stabilization (non-canonical pathway). Based on this data, we hypothesize that growth-factor induced EphA2 S897 phosphorylation contributes to the stabilization of the receptor by preserving it from degradation. Indeed, when mutating the S897 to alanine, which prevents phosphorylation and thereby is independent of ADAM17-mediated signaling, we could not anymore observe decreasing EphA2 protein levels after SCH772984 and TMI-005 treatment.

ADAM17 knockdown and/or inhibition of its activity resulted in strongly reduced total EphA2 protein levels, though transcription of EphA2 remained unaffected. Interestingly, cellular pre-treatment with recombinant human amphiregulin or the proteasome inhibitor MG-132 counteracted this observed decrease of total EphA2 protein levels in ADAM17 knockdown cells or in cells treated with TMI-005. These results suggest that ADAM17 stabilizes EphA2 protein levels on a post-translational level, potentially through ADAM17-released and growth-factor induced phosphorylation of EphA2 S897 thereby preventing its proteasomal degradation.

Interestingly, we also observed a secondary mechanism, by which ADAM17 regulates the protein levels of the EphA2 receptor. Our group and others have shown that recombinant ephrin-A1 Fc induces rapid internalization and proteasomal degradation of its receptor EphA2 (24). Immunofluorescent probing demonstrated that ADAM17 inhibition with TMI-005 induced a delayed but a similar internalization pattern as cellular treatment with recombinant, full-length ephrin-A1 Fc treatment. Thus, ADAM17 might also regulate EphA2 through modulating cleavage of the *trans*-acting membrane-bound ligand, ephrin-A1, thereby inactivating it as an internalization-inducing EphA2-ligand (canonical pathway). Indeed, we could detect significantly decreased levels of soluble ephrin-A1 in the supernatants, derived from TMI-005 or MEDI3622 treated NCI-H358 cells. These results corroborate that cleaved, soluble and monomeric ephrin-A1 does not result in EphA2 internalization (33–36). Together with the results of the acellular cleavage assay, we firstly describe ephrin-A1 as a novel direct substrate of ADAM17. To follow up on these results, it will be of interest to investigate to which extent ephrin-A1 is present on ADAM17-inhibited cells and whether short-term autophosphorylation effects can be determined on EphA2 tyrosine residues.

**Figure 6:**
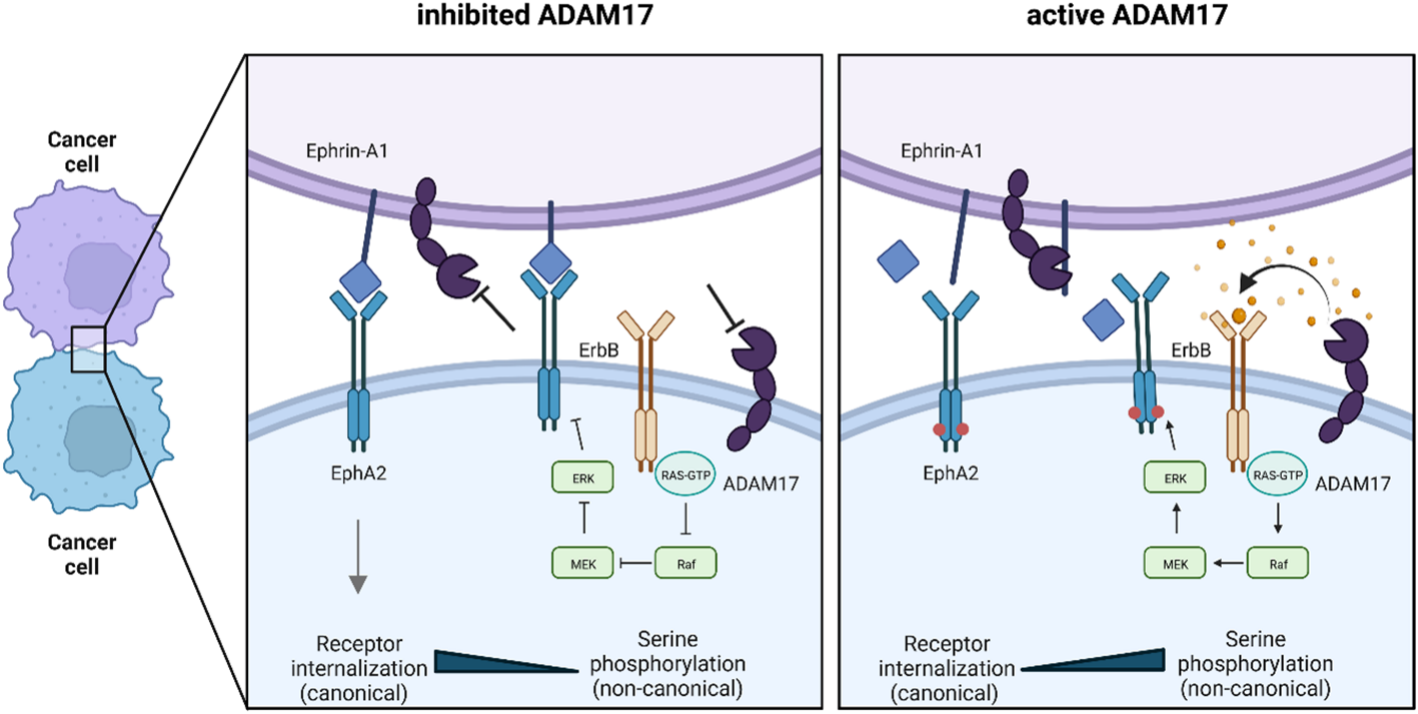
Graphical abstract: The dual role of ADAM17 on EphA2 signaling. Based on our data, ADAM17 influences EphA2 signaling by two mechanisms: 1) Release of growth factors that act on EphA2 pS897 *via* the MAPK pathway and thereby activate the non-canonical pathway. 2) Cleavage of GPI-anchored ephrin-A1 to prevent receptor internalization and thereby blocking the canonical signaling pathway. In red: EphA2 S897 phosphorylation.

Based on our data we postulate two mechanisms by which ADAM17 influences both the canonical and non-canonical pathway of EphA2 and ultimately contributes to oncogenic cellular behavior (**Figure 6**).

First, the non-canonical pathway of EphA2 depends on growth factors that stimulate other RTKs and their downstream kinases that catalyze EphA2 S897 phosphorylation. ADAM17 cleaves a majority of these factors, such as amphiregulin and other EGF family ligands, and thereby contributes to these signaling pathways (20, 26). As the non-canonical pathway is considered oncogenic, ADAM17 mediates EphA2 pS897-dependent tumor growth and (IR-induced) cell migration. Second, the canonical pathway requires direct cell-cell interaction for EphA2/ephrin-A1 binding, after which the complex is internalized (33–35). The resulting downstream signaling inhibits oncogenic pathways, such as the MAPK pathway (9). In cancer cells, where ADAM17 is highly expressed and active, ADAM17 directly cleaves ephrin-A1 and thereby disrupts EphA2/ephrin-A1 binding. Eventually, this results in the inhibition of the canonical, tumor-suppressive pathway.

Overall, these results support our understanding of ADAM17 and its multitude of regulated downstream processes as a relevant target to overcome intrinsic and acquired radiation resistance. At the same time, we demonstrate to the best of our knowledge for the first time a mechanistic link between ADAM17 and the EphA2-axis in the context of ionizing radiation-induced cell migration.

Although many have studied the increased migratory and invasive capabilities of various cancer cell lines after irradiation, it is challenging to identify and target the molecular drivers of IR-induced cell dissemination. Here, we illustrate the importance of ADAM17 for cell migration in the context of putative metastasis formation through EphA2 pS897-mediated signaling. Targeting a bottleneck like ADAM17 can affect the entire composition of the TME and tumor growth, angiogenesis, metastasis, and invasion. Importantly, these are all processes, which are profoundly triggered by radiotherapy. Conclusively we advocate that foremost combined treatment strategies together with radiotherapy (e.g. with anti-ADAM17 agents), rather than monotherapies will advance our collective battle against cancer.

## Data availability statement

The original contributions presented in the study are included in the article/**Supplementary Material**. Further inquiries can be directed to the corresponding author.

## Author contributions

VW: Investigation, methodology, writing-original draft, project administration, writing-review and editing. FT: Methodology. RW: Methodology. MP: Conceptualization, resources, supervision, funding acquisition, writing-original draft, project administration, writing-review and editing. All authors contributed to the article and approved the submitted version.
