# Supplementary material for: The role of EphA2 in ADAM17- and ionizing radiation-enhanced lung cancer cell migration
Source: Front Oncol. 2023 Mar 14;13:1117326. doi: 10.3389/fonc.2023.1117326 (PMC10043294; doi:10.3389/fonc.2023.1117326)
Supplement: Supplementary file 1 [file DataSheet_1.docx]

Supplementary Material

**The Role of EphA2 in ADAM17- and Ionizing Radiation-Enhanced Cancer Cell Migration**

Verena Waller, Fabienne Tschanz, Rona Winkler, and Martin Pruschy

*** Correspondence:**

Corresponding Author: martin.pruschy@uzh.ch

**Supplementary Figure 1.**


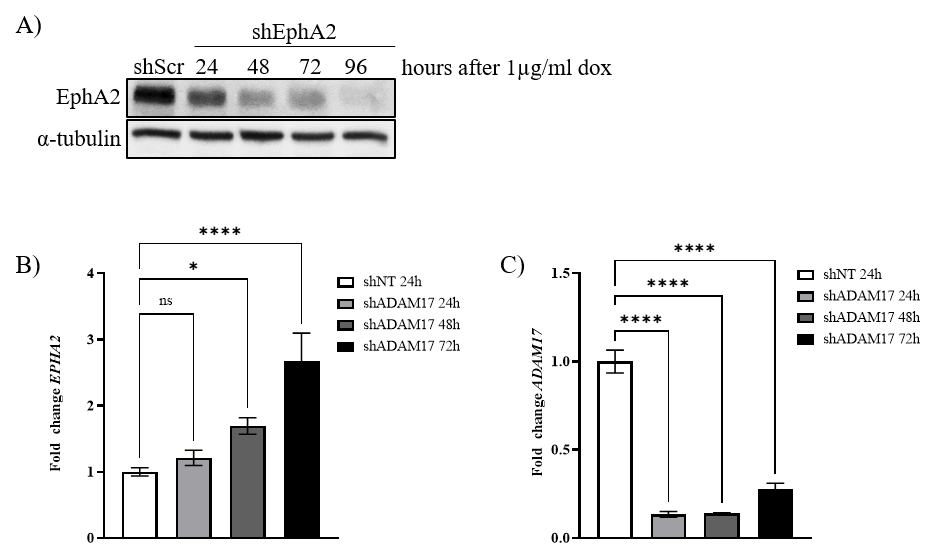


**Supplementary Figure S1:** (A) EphA2 knockdown in NCI-H358 cells over time after doxycycline induction (1µg/ml). (B) ADAM17 knockdown increases *EphA2* mRNA levels over time. This effect was not further investigated, as we could not identify the same increase on the protein level. (C) ADAM17 knockdown abrogated *ADAM17* gene expression. Bar graphs represent average EphA2/ADAM17 mRNA expression relative to the housekeeping control GAPDH ± SEM from two independent biological replicates; ns, non-significant, *, P < 0.05; **, P < 0.01; ***, P < 0.001; ****, P < 0.0001.

**Supplementary Figure 2.**


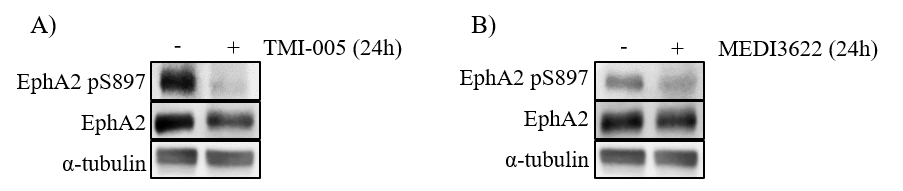


**Supplementary Figure S2:** EphA2 pS897 and total EphA2 protein levels in PC-3 cells were reduced upon (A) TMI-005 (25µM) and (B) MEDI3622 (200nM) treatment.

**Supplementary Figure 3.**


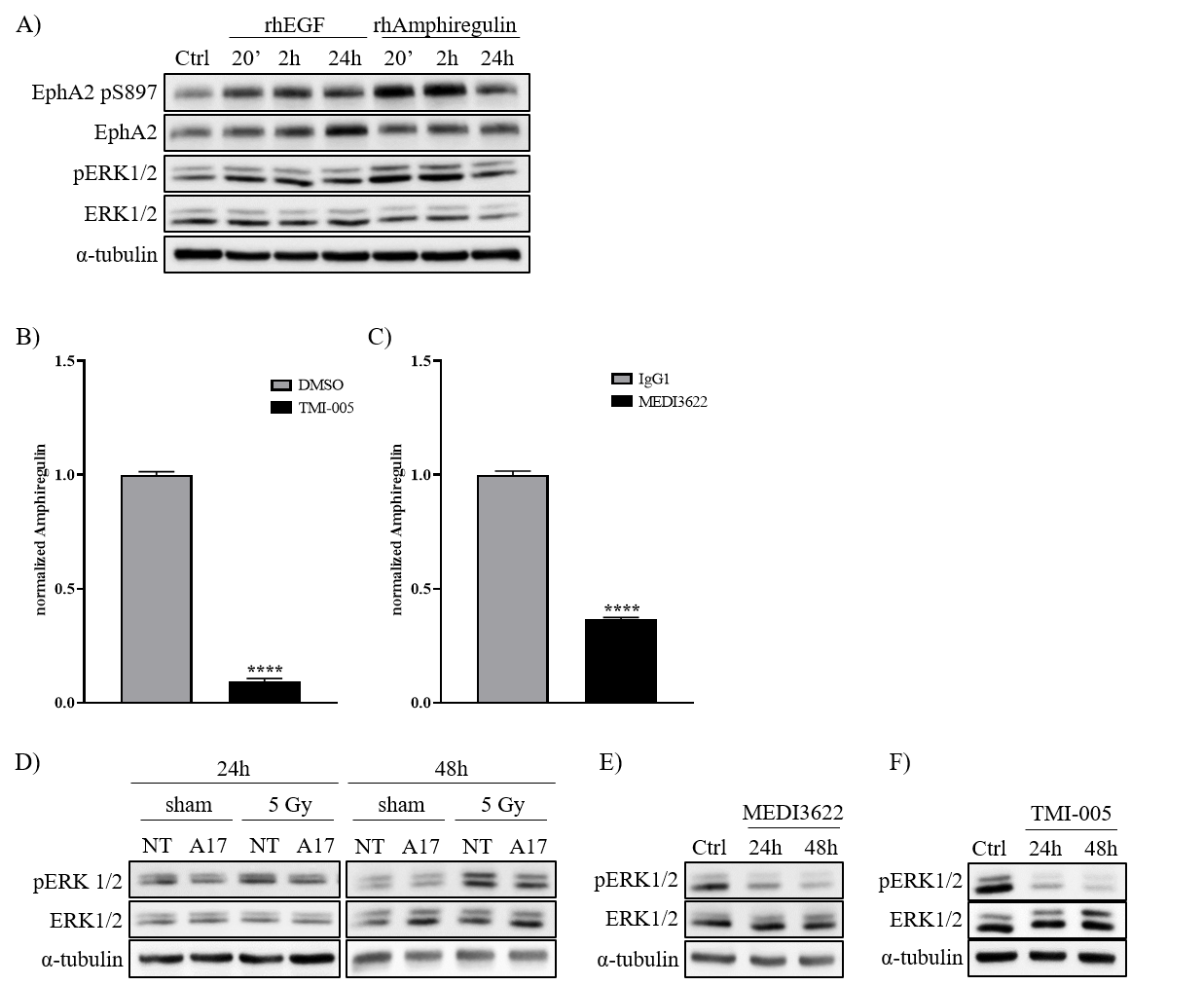


**Supplementary Figure S3:** (A) rhEGF and rhAmphiregulin increase EphA2 S897 phosphorylation levels at different time points. (B) and (C) Amphiregulin release from NCI-H358 cells was determined via ELISA. Amphiregulin release was reduced after ADAM17 inhibition with (B) TMI-005 and (C) MEDI3622. ERK1/2 phosphorylation was (D) increased after IR (5 Gy) and reduced in ADAM17-deficient cells, (E) TMI-005 treated and (F) MEDI3622 treated cells. Bar graphs represent amphiregrulin concentration normalized to total cellular protein amount ± SEM from three independent biological replicates. *, P < 0.05; **, P < 0.01; ***, P < 0.001; ****, P < 0.0001.

**Supplementary Figure 4.**


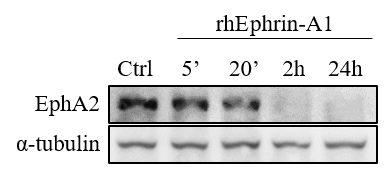


**Supplementary Figure S4:** rhEprhin-A1 Fc treatment leads to the degradation of EphA2 after 2h.

**Supplementary Figure 5.**


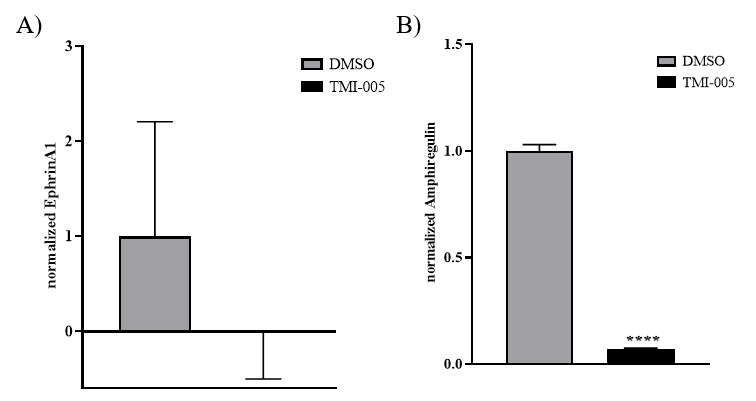


**Supplementary Figure S5:** ELISA of PC-3 derived supernatants against endogenous, soluble (A) ephrin-A1 and (B) amphiregulin. TMI-005 treatment reduced release of both ligands. Bar graphs represent ephrin-A1/amphiregulin concentrations normalized to the total cellular protein amount ± SEM from two independent biological replicates. *, P < 0.05; **, P < 0.01; ***, P < 0.001; ****, P < 0.0001.
